# 5-(4-Fluoro­phen­yl)-3-methyl­sulfanyl-2-phenyl-1-benzofuran

**DOI:** 10.1107/S1600536811036713

**Published:** 2011-09-30

**Authors:** Hong Dae Choi, Pil Ja Seo, Byeng Wha Son, Uk Lee

**Affiliations:** a Department of Chemistry, Dongeui University, San 24 Kaya-dong Busanjin-gu, Busan 614-714, Republic of Korea; b Department of Chemistry, Pukyong National University, 599-1 Daeyeon 3-dong, Nam-gu, Busan 608-737, Republic of Korea

## Abstract

In the title compound, C_21_H_15_FOS, the dihedral angle between the mean plane of the benzofuran fragment and the mean planes of the pendant 4-fluoro­benzene and phenyl rings are 31.72 (6)° and 32.51 (6)°, respectively. In the crystal, the mol­ecules are linked by weak C—H⋯π inter­actions. The crystal studied was a merohedral twin with a 0.62 (9):0.38 (9) domain ratio.

## Related literature

For background to the pharmacological activity of benzofuran compounds, see: Aslam *et al.* (2009 ▶); Galal *et al.* (2009 ▶); Khan *et al.* (2005 ▶). For natural products with benzofuran rings, see: Akgul & Anil (2003 ▶); Soekamto *et al.* (2003 ▶). For structural studies of 2-(4-halophen­yl)-3-methyl­sulfanyl-5-phenyl-1-benzo­furan drivatives, see: Choi *et al.* (2009 ▶, 2010 ▶).
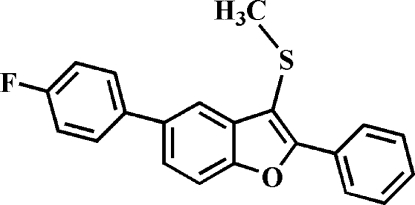


## Experimental

### 

#### Crystal data

C_21_H_15_FOS
*M* *_r_* = 334.39
Monoclinic, 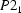

*a* = 10.6439 (15) Å
*b* = 7.2006 (10) Å
*c* = 11.7226 (17) Å
β = 115.396 (2)°
*V* = 811.6 (2) Å^3^
*Z* = 2
Mo *K*α radiation
μ = 0.21 mm^−1^
*T* = 173 K
0.36 × 0.29 × 0.10 mm

#### Data collection

Bruker SMART APEXII CCD diffractometer
Absorption correction: multi-scan (*SADABS*; Bruker, 2009 ▶) *T*_min_ = 0.928, *T*_max_ = 0.979
7372 measured reflections
3194 independent reflections
2540 reflections with *I* > 2σ(*I*)
*R*_int_ = 0.035

#### Refinement

*R*[*F* ^2^ > 2σ(*F* ^2^)] = 0.038
*wR*(*F* ^2^) = 0.106
*S* = 1.10
3194 reflections
219 parameters
1 restraint
H-atom parameters constrained
Δρ_max_ = 0.21 e Å^−3^
Δρ_min_ = −0.20 e Å^−3^
Absolute structure: Flack (1983 ▶), 1278 Friedel pairs
Flack parameter: 0.38 (9)

### 

Data collection: *APEX2* (Bruker, 2009 ▶); cell refinement: *SAINT* (Bruker, 2009 ▶); data reduction: *SAINT*; program(s) used to solve structure: *SHELXS97* (Sheldrick, 2008 ▶); program(s) used to refine structure: *SHELXL97* (Sheldrick, 2008 ▶); molecular graphics: *ORTEP-3* (Farrugia, 1997 ▶) and *DIAMOND* (Brandenburg, 1998 ▶); software used to prepare material for publication: *SHELXL97*.

## Supplementary Material

Crystal structure: contains datablock(s) global, I. DOI: 10.1107/S1600536811036713/hb6402sup1.cif

Structure factors: contains datablock(s) I. DOI: 10.1107/S1600536811036713/hb6402Isup2.hkl

Supplementary material file. DOI: 10.1107/S1600536811036713/hb6402Isup3.cml

Additional supplementary materials:  crystallographic information; 3D view; checkCIF report

## Figures and Tables

**Table 1:** Hydrogen-bond geometry (Å, °) *Cg* is the centroid of the C15–C20 phenyl ring.

*D*—H⋯*A*	*D*—H	H⋯*A*	*D*⋯*A*	*D*—H⋯*A*
C14—H14⋯*Cg*^i^	0.95	2.76	3.448 (2)	130

Symmetry code: (i) 
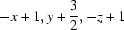
.

**Fig. 1.**
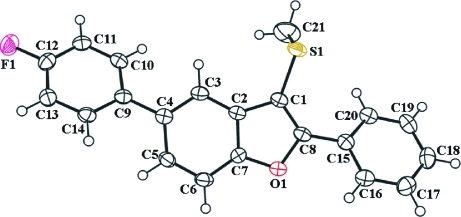
The molecular structure of the title compound with displacement ellipsoids drawn at the 50% probability level. H atoms are presented as small spheres of arbitrary radius.

**Fig. 2.**
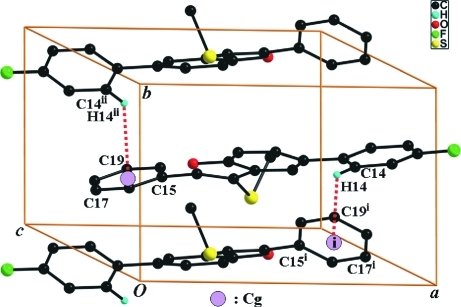
A view of the C—H···π interactions (dotted lines) in the crystal structure of the title compound. Symmetry codes: (i) - x + 1, y - 1/2, - z + 1; (ii) - x + 1, y + 1/2, - z + 1.]

## supplementary crystallographic information

### Comment

Recently, many compounds having a benzofuran moiety have drawn much attention due to their valuable pharmacological properties such as antibacterial and antifungal, antitumor and antiviral, and antimicrobial activities (Aslam *et al.*, 2009, Galal *et al.*, 2009, Khan *et al.*, 2005). These benzofuran derivatives occur in a wide range of natural products (Akgul & Anil, 2003; Soekamto *et al.*, 2003). As a part of our ongoing studies of the substituent effect on the solid state structures of 2-(4-halophenyl)-3-methylsulfanyl-5-phenyl-1-benzofuran analogues (Choi *et al.*, 2009, 2010), we report herein the crystal structure of the title compound.

The title compound crystallizes as the non-centrosymmetric space group *P*_21_ in spite of having no asymmetric C atoms. The crystal studied was an inversion twin with a 0.68 (9) : 0.32 (9) domain ratio.

In the title molecule (Fig. 1), the benzofuran unit is essentially planar, with a mean deviation of 0.006 (2) Å from the least-squares plane defined by the nine constituent atoms. The dihedral angle formed by the 4-fluorophenyl ring and the mean plane of the benzofurn fragment is 31.72 (6)°. The dihedral angle between the phenyl ring and the mean plane of the benzofurn fragment is 32.51 (6)°. The crystal packing (Fig. 2) is stabilized by intermolecular C—H···π interactions between a 4-fluorophenyl H atom and the phenyl ring (Table 1; C14—H14···Cg^i^, Cg is the centroid of the C15–C20 phenyl ring).

### Experimental

Zinc chloride (273 mg, 2.0 mmol) was added to a stirred solution of 4-fluoro-4'-hydroxybiphenyl (376 mg, 2.0 mmol) and 2-chloro-2-methylsulfanylacetophenone (401 mg, 2.0 mmol) in dichloromethane (30 mL) at room temperature, and stirring was continued at the same temperature for 1 h. The reaction was quenched by the addition of water and the organic layer was separated, dried over magnesium sulfate, filtered and concentrated at reduced pressure. The residue was purified by column chromatography (hexane–benzene, 5:2 v/v) to afford the title compound as a colorless solid [yield 61%, m.p. 415–416 K; *R*_f_ = 0.78 (hexane–benzene, 5:2 v/v)]. Colourless blocks were prepared by slow evaporation of a solution of the title compound in carbon tetrachloride at room temperature.

### Refinement

The reported Flack parameter was obtained by TWIN/BASF procedure in SHELXL (Sheldrick, 2008). All H atoms were positioned geometrically and refined using a riding model, with C—H = 0.95 Å  for aryl and  0.98 Å for methyl H atoms. *U*_iso_(H) =1.2*U*_eq_(C) for aryl  and 1.5*U*_eq_(C) for methyl H atoms.

### Crystal data

**Table:**

C_21_H_15_FOS	*F*(000) = 348
*M**_r_* = 334.39	*D*_x_ = 1.368 Mg m^−^^3^
Monoclinic, *P*2_1_	Mo *K*α radiation, λ = 0.71073 Å
Hall symbol: P 2yb	Cell parameters from 2652 reflections
*a* = 10.6439 (15) Å	θ = 3.4–26.6°
*b* = 7.2006 (10) Å	µ = 0.21 mm^−^^1^
*c* = 11.7226 (17) Å	*T* = 173 K
β = 115.396 (2)°	Block, colourless
*V* = 811.6 (2) Å^3^	0.36 × 0.29 × 0.10 mm
*Z* = 2	

### Data collection

**Table:**

Bruker SMART APEXII CCD diffractometer	3194 independent reflections
Radiation source: rotating anode	2540 reflections with *I* > 2σ(*I*)
graphite multilayer	*R*_int_ = 0.035
Detector resolution: 10.0 pixels mm^-1^	θ_max_ = 27.0°, θ_min_ = 1.9°
φ and ω scans	*h* = −12→13
Absorption correction: multi-scan (*SADABS*; Bruker, 2009)	*k* = −8→9
*T*_min_ = 0.928, *T*_max_ = 0.979	*l* = −14→14
7372 measured reflections	

### Refinement

**Table:**

Refinement on *F*^2^	Secondary atom site location: difference Fourier map
Least-squares matrix: full	Hydrogen site location: difference Fourier map
*R*[*F*^2^ > 2σ(*F*^2^)] = 0.038	H-atom parameters constrained
*wR*(*F*^2^) = 0.106	*w* = 1/[σ^2^(*F*_o_^2^) + (0.0545*P*)^2^] where *P* = (*F*_o_^2^ + 2*F*_c_^2^)/3
*S* = 1.10	(Δ/σ)_max_ < 0.001
3194 reflections	Δρ_max_ = 0.21 e Å^−^^3^
219 parameters	Δρ_min_ = −0.20 e Å^−^^3^
1 restraint	Absolute structure: Flack (1983), 1278 Friedel pairs
Primary atom site location: structure-invariant direct methods	Flack parameter: 0.38 (9)

### Special details

**Table:**

Geometry. All esds (except the esd in the dihedral angle between two l.s. planes) are estimated using the full covariance matrix. The cell esds are taken into account individually in the estimation of esds in distances, angles and torsion angles; correlations between esds in cell parameters are only used when they are defined by crystal symmetry. An approximate (isotropic) treatment of cell esds is used for estimating esds involving l.s. planes.
Refinement. Refinement of F^2^ against ALL reflections. The weighted R-factor wR and goodness of fit S are based on F^2^, conventional R-factors R are based on F, with F set to zero for negative F^2^. The threshold expression of F^2^ > 2sigma(F^2^) is used only for calculating R-factors(gt) etc. and is not relevant to the choice of reflections for refinement. R-factors based on F^2^ are statistically about twice as large as those based on F, and R- factors based on ALL data will be even larger.

### Fractional atomic coordinates and isotropic or equivalent isotropic displacement parameters (Å^2^)

**Table:**

	*x*	*y*	*z*	*U*_iso_*/*U*_eq_	
S1	0.42262 (6)	0.39699 (11)	0.12213 (5)	0.03595 (18)	
F1	1.34923 (15)	0.4759 (3)	0.74321 (14)	0.0487 (4)	
O1	0.34169 (16)	0.4817 (2)	0.41757 (13)	0.0299 (4)	
C1	0.4191 (2)	0.4505 (3)	0.26649 (19)	0.0269 (5)	
C2	0.5384 (2)	0.4604 (3)	0.38747 (19)	0.0259 (5)	
C3	0.6812 (2)	0.4542 (3)	0.42791 (19)	0.0280 (5)	
H3	0.7195	0.4416	0.3685	0.034*	
C4	0.7681 (2)	0.4667 (3)	0.5565 (2)	0.0272 (5)	
C5	0.7074 (3)	0.4836 (4)	0.6427 (2)	0.0306 (5)	
H5	0.7670	0.4914	0.7303	0.037*	
C6	0.5661 (3)	0.4890 (4)	0.6047 (2)	0.0327 (6)	
H6	0.5269	0.4998	0.6635	0.039*	
C7	0.4842 (2)	0.4781 (4)	0.47627 (19)	0.0273 (5)	
C8	0.3042 (2)	0.4667 (3)	0.28925 (19)	0.0272 (5)	
C9	0.9222 (2)	0.4655 (3)	0.60428 (19)	0.0264 (5)	
C10	0.9871 (3)	0.5412 (3)	0.5334 (2)	0.0294 (5)	
H10	0.9317	0.5924	0.4526	0.035*	
C11	1.1301 (3)	0.5429 (3)	0.5785 (2)	0.0307 (5)	
H11	1.1734	0.5928	0.5294	0.037*	
C12	1.2080 (2)	0.4703 (4)	0.6967 (2)	0.0329 (5)	
C13	1.1500 (2)	0.3955 (4)	0.7699 (2)	0.0330 (5)	
H13	1.2067	0.3478	0.8515	0.040*	
C14	1.0067 (2)	0.3910 (4)	0.72242 (18)	0.0290 (5)	
H14	0.9648	0.3359	0.7712	0.035*	
C15	0.1550 (2)	0.4714 (3)	0.2092 (2)	0.0274 (5)	
C16	0.0615 (2)	0.4006 (4)	0.25293 (19)	0.0312 (5)	
H16	0.0956	0.3464	0.3346	0.037*	
C17	−0.0797 (3)	0.4087 (4)	0.1787 (2)	0.0373 (6)	
H17	−0.1424	0.3593	0.2090	0.045*	
C18	−0.1305 (3)	0.4888 (4)	0.0595 (2)	0.0383 (6)	
H18	−0.2278	0.4939	0.0082	0.046*	
C19	−0.0387 (3)	0.5614 (4)	0.0158 (2)	0.0359 (6)	
H19	−0.0736	0.6180	−0.0651	0.043*	
C20	0.1025 (3)	0.5520 (3)	0.0887 (2)	0.0316 (5)	
H20	0.1646	0.6002	0.0573	0.038*	
C21	0.4698 (4)	0.6154 (6)	0.0774 (3)	0.0679 (11)	
H21A	0.3992	0.7088	0.0688	0.102*	
H21B	0.4756	0.6015	−0.0033	0.102*	
H21C	0.5602	0.6553	0.1425	0.102*	

### Atomic displacement parameters (Å^2^)

**Table:**

	*U*^11^	*U*^22^	*U*^33^	*U*^12^	*U*^13^	*U*^23^
S1	0.0428 (4)	0.0435 (3)	0.0252 (2)	0.0018 (3)	0.0181 (2)	−0.0041 (3)
F1	0.0285 (9)	0.0664 (11)	0.0510 (9)	−0.0028 (8)	0.0168 (7)	0.0080 (8)
O1	0.0319 (9)	0.0355 (8)	0.0257 (7)	0.0000 (8)	0.0157 (6)	−0.0012 (7)
C1	0.0348 (13)	0.0234 (11)	0.0242 (10)	0.0000 (10)	0.0143 (9)	−0.0008 (8)
C2	0.0336 (13)	0.0234 (10)	0.0236 (9)	0.0006 (10)	0.0152 (9)	−0.0005 (8)
C3	0.0348 (13)	0.0264 (11)	0.0271 (10)	0.0023 (10)	0.0175 (9)	−0.0009 (9)
C4	0.0334 (13)	0.0216 (9)	0.0287 (10)	−0.0003 (11)	0.0152 (9)	−0.0002 (9)
C5	0.0336 (14)	0.0354 (12)	0.0225 (10)	−0.0029 (11)	0.0117 (10)	−0.0021 (9)
C6	0.0386 (15)	0.0375 (14)	0.0282 (11)	−0.0013 (12)	0.0204 (10)	−0.0037 (10)
C7	0.0253 (13)	0.0301 (11)	0.0281 (11)	0.0002 (11)	0.0130 (9)	−0.0002 (9)
C8	0.0362 (13)	0.0251 (10)	0.0224 (9)	0.0001 (11)	0.0144 (9)	0.0016 (8)
C9	0.0324 (14)	0.0223 (10)	0.0280 (10)	−0.0022 (11)	0.0162 (10)	−0.0031 (9)
C10	0.0371 (15)	0.0270 (11)	0.0250 (10)	−0.0001 (10)	0.0142 (10)	0.0011 (9)
C11	0.0354 (15)	0.0297 (12)	0.0338 (12)	−0.0021 (11)	0.0213 (11)	0.0003 (10)
C12	0.0273 (14)	0.0327 (12)	0.0400 (12)	−0.0026 (12)	0.0157 (10)	−0.0038 (11)
C13	0.0309 (13)	0.0363 (12)	0.0305 (10)	0.0000 (13)	0.0119 (9)	0.0017 (11)
C14	0.0339 (13)	0.0289 (10)	0.0281 (9)	−0.0041 (12)	0.0170 (9)	0.0024 (11)
C15	0.0315 (13)	0.0221 (10)	0.0289 (10)	0.0027 (11)	0.0131 (9)	−0.0017 (9)
C16	0.0389 (14)	0.0256 (10)	0.0321 (10)	0.0011 (12)	0.0181 (10)	0.0023 (11)
C17	0.0392 (14)	0.0305 (13)	0.0449 (12)	−0.0061 (12)	0.0208 (11)	−0.0044 (12)
C18	0.0296 (14)	0.0326 (14)	0.0441 (14)	0.0015 (12)	0.0076 (11)	−0.0083 (11)
C19	0.0436 (17)	0.0294 (13)	0.0266 (11)	0.0027 (12)	0.0073 (11)	−0.0005 (9)
C20	0.0387 (15)	0.0273 (12)	0.0303 (11)	−0.0009 (11)	0.0162 (11)	0.0000 (9)
C21	0.091 (3)	0.073 (2)	0.0554 (19)	−0.026 (2)	0.047 (2)	0.0069 (16)

### Geometric parameters (Å, °)

**Table:**

S1—C1	1.752 (2)	C10—H10	0.9500
S1—C21	1.796 (3)	C11—C12	1.377 (3)
F1—C12	1.362 (3)	C11—H11	0.9500
O1—C7	1.371 (3)	C12—C13	1.365 (3)
O1—C8	1.385 (2)	C13—C14	1.382 (3)
C1—C8	1.364 (3)	C13—H13	0.9500
C1—C2	1.444 (3)	C14—H14	0.9500
C2—C3	1.385 (3)	C15—C16	1.397 (3)
C2—C7	1.396 (3)	C15—C20	1.402 (3)
C3—C4	1.393 (3)	C16—C17	1.378 (3)
C3—H3	0.9500	C16—H16	0.9500
C4—C5	1.418 (3)	C17—C18	1.388 (4)
C4—C9	1.489 (3)	C17—H17	0.9500
C5—C6	1.374 (4)	C18—C19	1.385 (4)
C5—H5	0.9500	C18—H18	0.9500
C6—C7	1.381 (3)	C19—C20	1.376 (3)
C6—H6	0.9500	C19—H19	0.9500
C8—C15	1.458 (3)	C20—H20	0.9500
C9—C14	1.395 (3)	C21—H21A	0.9800
C9—C10	1.399 (3)	C21—H21B	0.9800
C10—C11	1.380 (3)	C21—H21C	0.9800
			
C1—S1—C21	102.33 (13)	C10—C11—H11	120.9
C7—O1—C8	106.56 (16)	F1—C12—C13	118.6 (2)
C8—C1—C2	106.77 (18)	F1—C12—C11	118.5 (2)
C8—C1—S1	126.90 (17)	C13—C12—C11	122.9 (2)
C2—C1—S1	125.83 (17)	C12—C13—C14	118.4 (2)
C3—C2—C7	119.45 (19)	C12—C13—H13	120.8
C3—C2—C1	135.12 (19)	C14—C13—H13	120.8
C7—C2—C1	105.4 (2)	C13—C14—C9	121.4 (2)
C2—C3—C4	119.4 (2)	C13—C14—H14	119.3
C2—C3—H3	120.3	C9—C14—H14	119.3
C4—C3—H3	120.3	C16—C15—C20	118.8 (2)
C3—C4—C5	118.9 (2)	C16—C15—C8	120.38 (19)
C3—C4—C9	121.32 (19)	C20—C15—C8	120.8 (2)
C5—C4—C9	119.82 (19)	C17—C16—C15	120.6 (2)
C6—C5—C4	122.8 (2)	C17—C16—H16	119.7
C6—C5—H5	118.6	C15—C16—H16	119.7
C4—C5—H5	118.6	C16—C17—C18	120.1 (2)
C5—C6—C7	116.3 (2)	C16—C17—H17	119.9
C5—C6—H6	121.8	C18—C17—H17	119.9
C7—C6—H6	121.8	C19—C18—C17	119.8 (2)
O1—C7—C6	126.3 (2)	C19—C18—H18	120.1
O1—C7—C2	110.47 (18)	C17—C18—H18	120.1
C6—C7—C2	123.3 (2)	C20—C19—C18	120.5 (2)
C1—C8—O1	110.74 (19)	C20—C19—H19	119.8
C1—C8—C15	134.14 (19)	C18—C19—H19	119.8
O1—C8—C15	115.12 (19)	C19—C20—C15	120.2 (2)
C14—C9—C10	117.8 (2)	C19—C20—H20	119.9
C14—C9—C4	120.84 (19)	C15—C20—H20	119.9
C10—C9—C4	121.3 (2)	S1—C21—H21A	109.5
C11—C10—C9	121.4 (2)	S1—C21—H21B	109.5
C11—C10—H10	119.3	H21A—C21—H21B	109.5
C9—C10—H10	119.3	S1—C21—H21C	109.5
C12—C11—C10	118.1 (2)	H21A—C21—H21C	109.5
C12—C11—H11	120.9	H21B—C21—H21C	109.5
			
C21—S1—C1—C8	107.9 (3)	C3—C4—C9—C14	−149.0 (2)
C21—S1—C1—C2	−81.3 (2)	C5—C4—C9—C14	31.9 (3)
C8—C1—C2—C3	−179.5 (3)	C3—C4—C9—C10	31.9 (3)
S1—C1—C2—C3	8.2 (4)	C5—C4—C9—C10	−147.2 (2)
C8—C1—C2—C7	1.3 (3)	C14—C9—C10—C11	−0.2 (4)
S1—C1—C2—C7	−171.00 (18)	C4—C9—C10—C11	178.9 (2)
C7—C2—C3—C4	−0.3 (3)	C9—C10—C11—C12	−0.9 (3)
C1—C2—C3—C4	−179.4 (3)	C10—C11—C12—F1	−178.3 (2)
C2—C3—C4—C5	0.6 (3)	C10—C11—C12—C13	0.7 (4)
C2—C3—C4—C9	−178.4 (2)	F1—C12—C13—C14	179.7 (2)
C3—C4—C5—C6	−0.3 (4)	C11—C12—C13—C14	0.7 (4)
C9—C4—C5—C6	178.8 (2)	C12—C13—C14—C9	−1.9 (4)
C4—C5—C6—C7	−0.3 (4)	C10—C9—C14—C13	1.7 (4)
C8—O1—C7—C6	−179.9 (2)	C4—C9—C14—C13	−177.4 (2)
C8—O1—C7—C2	−0.2 (3)	C1—C8—C15—C16	148.6 (3)
C5—C6—C7—O1	−179.7 (2)	O1—C8—C15—C16	−31.5 (3)
C5—C6—C7—C2	0.7 (4)	C1—C8—C15—C20	−33.4 (4)
C3—C2—C7—O1	180.0 (2)	O1—C8—C15—C20	146.6 (2)
C1—C2—C7—O1	−0.7 (3)	C20—C15—C16—C17	0.4 (4)
C3—C2—C7—C6	−0.3 (4)	C8—C15—C16—C17	178.5 (3)
C1—C2—C7—C6	179.0 (2)	C15—C16—C17—C18	−0.5 (4)
C2—C1—C8—O1	−1.5 (3)	C16—C17—C18—C19	−0.3 (4)
S1—C1—C8—O1	170.70 (16)	C17—C18—C19—C20	1.0 (4)
C2—C1—C8—C15	178.5 (2)	C18—C19—C20—C15	−1.1 (4)
S1—C1—C8—C15	−9.3 (4)	C16—C15—C20—C19	0.3 (4)
C7—O1—C8—C1	1.1 (3)	C8—C15—C20—C19	−177.7 (2)
C7—O1—C8—C15	−178.9 (2)		

### Hydrogen-bond geometry (Å, °)

**Table:**

Cg is the centroid of the C15–C20 phenyl ring.

**Table:**

*D*—H···*A*	*D*—H	H···*A*	*D*···*A*	*D*—H···*A*
C14—H14···Cg^i^	0.95	2.76	3.448 (2)	130

Symmetry codes: (i) −*x*+1, *y*+3/2, −*z*+1.
